# Identifying candidate *Aspergillus* pathogenicity factors by annotation frequency

**DOI:** 10.1186/s12866-020-02031-y

**Published:** 2020-11-11

**Authors:** Kayla K. Pennerman, Guohua Yin, Anthony E. Glenn, Joan W. Bennett

**Affiliations:** 1 United States Department of Agriculture, Toxicology and Mycotoxin Research Unit, Athens, GA 30605 USA; 2 Department of Plant Biology, Rutgers University, The State University of New Jersey, New Brunswick, NJ 08901 USA

**Keywords:** *Aspergillus*, Comparative gene annotation, Comparative protein annotation, Hexokinase, Pathogenicity factors

## Abstract

**Background:**

Members of the genus *Aspergillus* display a variety of lifestyles, ranging from saprobic to pathogenic on plants and/or animals. Increased genome sequencing of economically important members of the genus permits effective use of “-omics” comparisons between closely related species and strains to identify candidate genes that may contribute to phenotypes of interest, especially relating to pathogenicity. Protein-coding genes were predicted from 216 genomes of 12 *Aspergillus* species, and the frequencies of various structural aspects (exon count and length, intron count and length, GC content, and codon usage) and functional annotations (InterPro, Gene Ontology, and Kyoto Encyclopedia of Genes and Genomes terms) were compared.

**Results:**

Using principal component analyses, the three sets of functional annotations for each strain were clustered by species. The species clusters appeared to separate by pathogenicity on plants along the first dimensions, which accounted for over 20% of the variance. More annotations for genes encoding pectinases and secondary metabolite biosynthetic enzymes were assigned to phytopathogenic strains from species such as *Aspergillus flavus*. In contrast, *Aspergillus fumigatus* strains, which are pathogenic to animals but not plants, were assigned relatively more terms related to phosphate transferases, and carbohydrate and amino-sugar metabolism. Analyses of publicly available RNA-Seq data indicated that one *A. fumigatus* protein among 17 amino-sugar processing candidates, a hexokinase, was up-regulated during co-culturing with human immune system cells.

**Conclusion:**

Genes encoding hexokinases and other proteins of interest may be subject to future manipulations to further refine understanding of *Aspergillus* pathogenicity factors.

**Supplementary Information:**

The online version contains supplementary material available at 10.1186/s12866-020-02031-y.

## Background

Only a minority of fungi are pathogenic, mostly on plants, while the majority of fungal species are saprobes or mutualists [1]. Numerous studies have investigated the underlying genetics of fungal pathogenicity as well as the environmental, biological and pathogenic relevance of fungi to basic science and human affairs [2]. The ascomycetous genus *Aspergillus* provides an intriguing model for studying differentiation among opportunistic pathogens (both plant and animal) and saprobic decomposers. All members of this genus live largely as saprobes. However, several species are able to cause rots on living plant tissues and/or invasive aspergillosis in immunocompromised mammals [3–5]. Systemic aspergillosis is a life-threatening disease posing considerable public health and economic concerns. In recent decades, there has been a rise in the numbers of cases of invasive aspergillosis, likely due to an increase in immunosuppressive chemotherapy treatments, as well as organ and stem cell transplantations [6–8]. Invasive aspergillosis is usually caused by strains of *A. fumigatus*; less common *Aspergillus* causative agents are *A. fischeri*, *A. flavus*, *A. nidulans*, *A. niger* and *A. terreus* [9]. Human pathogenesis by *Aspergillus* species is complex and requires the normally saprotrophic *Aspergillus* spp. to adapt to the environment of the human lung [10]. Hospitalizations due to *Aspergillus* infections cost the United States an estimated 1.2 billion dollars annually [11]. One *Aspergillus* species, *A*. *sydowii*, is salt-tolerant pathogen of coral and humans that may become more destructive with continued global warming, along with other pathogenic species [12].

Opportunistic plant infections by *Aspergillus* species are also common after drought, insect damage or other environmental stresses. In particular, infection by *A. flavus* and *A. parasiticus* strains cause large economic losses in agriculture due to associated contamination with mycotoxins, notably aflatoxins. Aflatoxin contamination alone costs the United States 50 million to 1.7 billion dollars each year [13]. *A. flavus* is not only the primary causative agent of *Aspergillus* infections and aflatoxin contamination in crops, it is simultaneously the second most common cause of aspergillosis in human patients. In contrast, while *A. fumigatus* is the most common cause of human and veterinary aspergillosis, it is not known to cause disease in any plant host [14–16]. Essentially, *Aspergillus* pathogens can be grouped by those that may infect both plants and animals (phytopathogenic), and those that are not known to cause diseases in plants (non-phytopathogenic). This distinction is not sufficiently explored in published research to date.

Different infection strategies are required to cause disease in different hosts; thus, pathogenic species and strains must have genes that enable disease-causing infections and suppress resistance responses. The expression of relevant genes may be influenced by environmental conditions such as nutrient composition and response to host defenses [17]. Mammalian pathogens have to evade circulating immune system cells while the phytopathogens must be able to penetrate sturdy cell walls. Further, animal fungal pathogens preferentially disperse by hyphae or arthroconidia instead of conidia, are frequently dimorphic and often lack a known complete sexual cycle [18, 19]. Plant pathogens have hydrolytic enzymes to degrade cuticles and plant cell walls, and the ability to form appressoria [19]. Phytopathogenicity genes necessary for disease development include those that are involved in host recognition, signaling, secondary metabolite synthesis, cell wall integrity, appressorial formation, degradation of host cuticle and cell wall, uptake of nutrients and genes with unknown roles [20]. For both plant and animal pathogens, the ability to withstand abiotic stresses within the host environment, such as hypoxia, oxidative burst and mammalian body temperatures, are likely additional virulence factors [16, 19, 21–24].

In this study, multiple comparisons of genome annotations, largely represented by *A. flavus* and *A. fumigatus*, were used to address the discrepancy between the host ranges of phytopathogenic and non-phytopathogenic *Aspergillus* species. The recent substantial increase in genomic sequencing of filamentous fungi permits more focused comparisons among strains between and within species [25]. Both structural (high-throughput prediction of the composition and arrangement of physical motifs) and functional (high-throughput prediction of the biological uses of gene products) genomics are routinely performed on newly-sequenced genomes. Usually, genome size, GC content, number of predicted genes and predicted functions of those genes are reported in genome announcements. Biological interpretations of large data sets may uncover hitherto overlooked genes that define what a fungus can or cannot do. A consistent method of gene prediction across a set of genomes and genome annotations is a useful starting place to identify genes that contribute to phenotypes of interest. Such a method has the benefit of not requiring wet laboratory work, complementing transcriptomic or proteomic experiments that test predictions and quantify expression levels, and leading to new hypotheses for targeted gene manipulations.

The present study demonstrates the utility of this approach by looking for new pathogenicity genes within the *Aspergillus* genus. The identified gene candidates are corroborated by reports on the effects of *Aspergillus* deletion mutants on pathogenicity or virulence, and by published transcriptomic data. To the authors’ knowledge, this the first report of a method to search for genes contributing to a phenotype of interest by gene annotation frequencies across a genus. The long-term objective of our research is to develop a pipeline for extracting new, mycologically-relevant information from the wealth of genomics data stored in public databases. These bioinformatics findings will guide hypotheses that can be tested later via methods such as gene deletion. Our immediate goals were to 1) conduct a comparative analysis of annotations for 216 *Aspergillus* genomes, 2) identify previously-unidentified pathogenicity factors and 3) use previously-reported information to determine if the candidate pathogenicity factors are known to affect virulence or change expression level during infection processes.

## Results

### Functional annotation relative frequencies clustered strains by species

A total of 216 *Aspergillus* genomes were initially included in the study (Additional file 1). The structural aspects of predicted genes were similar regardless of host association (Additional files 2, 3, 4). The ranges are given in parentheses for the following attributes: gene counts (8833 to 14,749), average gene lengths (703.8 to 1815.7 bp), average exon counts (1.9 to 3.6), average exon lengths (279.2 to 540.8 bp), average intron counts (1.3 to 2.6) and average intron length (77.3 to 92.8 bp). GC content was lower in intronic sequences (32.3 to 40.8%) compared to exonic sequences (52.1 to 58.4%). According to ANOVA, the number of predicted genes and average gene GC content differed among the species (*p*-value < 2^− 16^). By *t*-test, there were no statistically significant differences between the species classified as phytopathogenic or not. Only *A*. *terreus* strains had significantly different exonic and gene GC content compared to all other species. Some codon bias in favor of AAG for lysine and GAG for glutamate instead of equivalent codons was observed (Additional file 3). The most frequent amino acids in the translated sequences were alanine, glycine, leucine and serine; the least frequent were cysteine and tryptophan (Additional file 4). A *t*-test indicated that non-phytopathogenic strains had significantly fewer (45.6 versus 79.5 average clusters, *p*-value = 8.8^− 14^) predicted secondary metabolite clusters (Additional file 5).

**Fig. 1:**
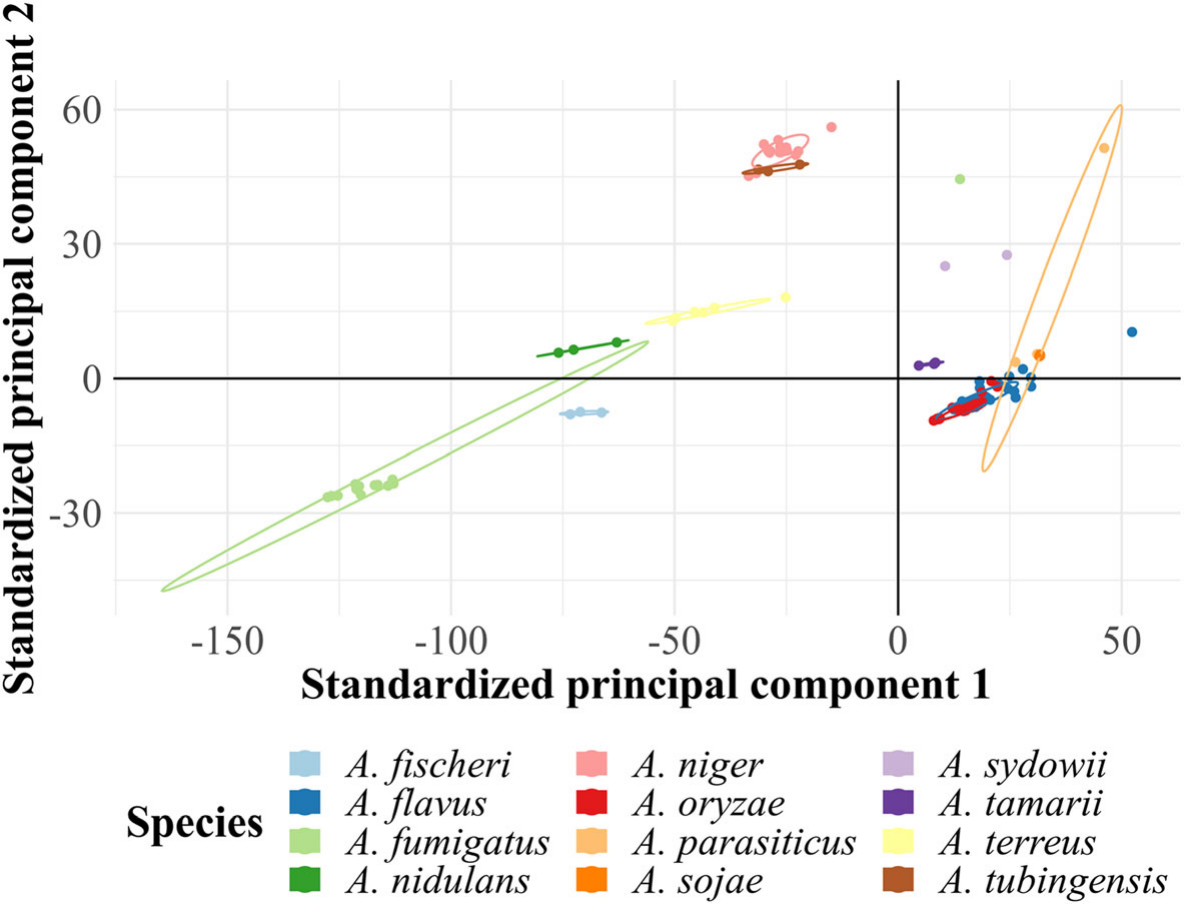
IPR functional annotation cluster by *Aspergillus* species. Non-phytopathogenic strains trend to the left of the plot. Other PCA plots and the corresponding scree plots are shown in Additional file 6

The first and second principal components of principal component analysis (PCA) results explained 73.3% of the variance among structural annotations and 22.3 to 38.7% of the variances of relative frequencies of functional annotations. Both structural and functional annotations yielded grouping of strains that largely clustered with other members of the same species (Fig. 1; Additional file 6). There was an unsurprising overlap of the closely-related *A. flavus*, *A*. *oryzae*, *A. parasiticus* and *A*. *sojae* species. *A. niger* and *A*. *tubingensis* strains clustered near each other as well. With the exceptions of phytopathogenic *A*. *terreus* and non-phytopathogenic *A*. *sydowii*, most of the species were separated by pathogenicity along the first principal. Use of the third to tenth principal components did not improve graphed separation. Strains *A*. *sydowii* BOBA1 and *A*. *terreus* T3_Kankrej were removed from PCA figures of functional annotations as they did not cluster with any other strain and increased the spans of the first and second principal components by more than 100% (Fig. 1; Additional file 6). The inclusion of the two strains did not greatly affect the PCA results using the structural annotations and were included in Additional file 6a. Due to the extreme outlying natures of strains BOBA1 and T3_Krankrej, they were not used in further analyses.

### Amino-sugar terms were assigned more frequently to non-phytopathogens

**Fig. 2:**
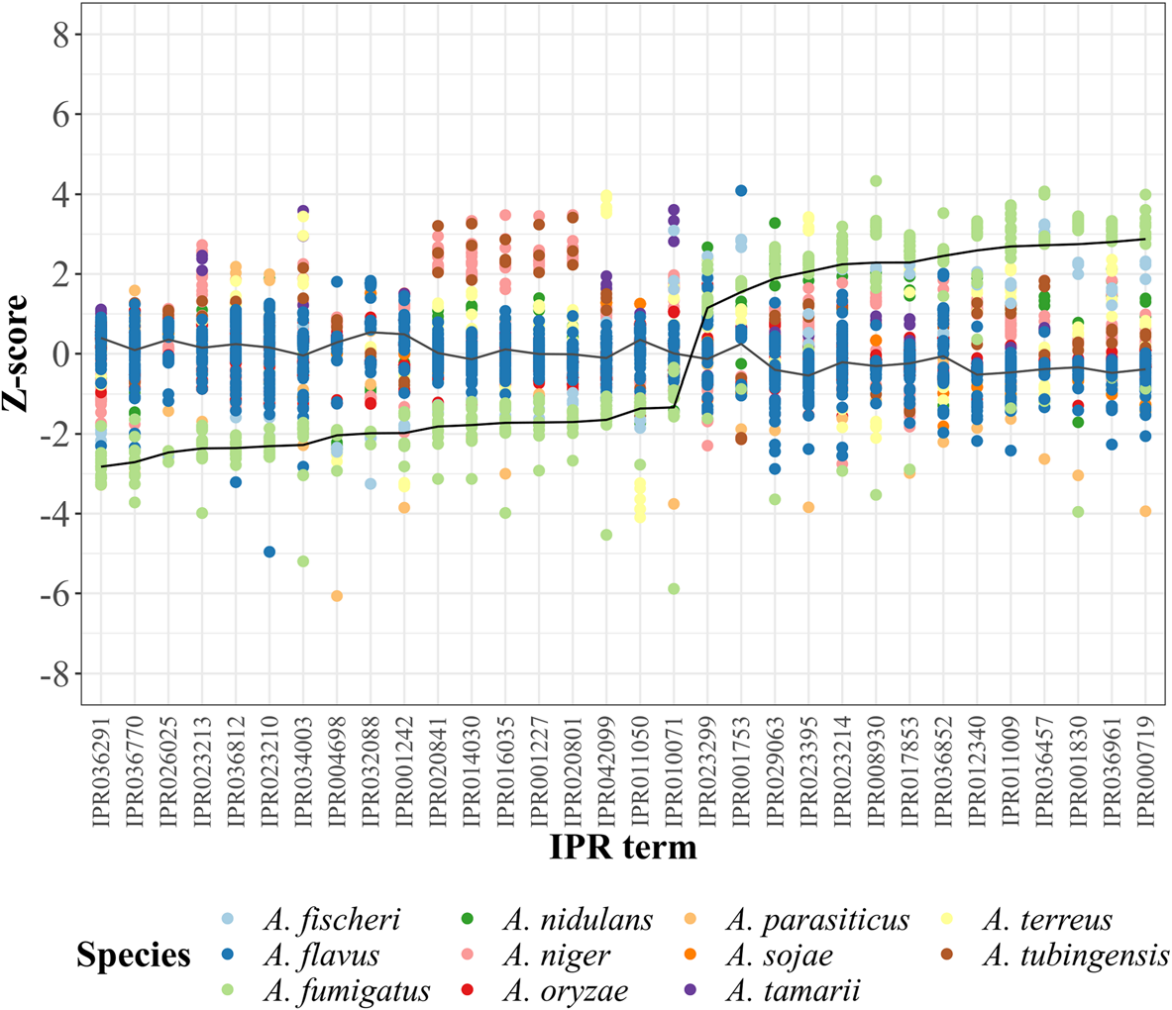
Relative frequencies of IPR terms associated with pathogenicity and/or virulence. Only annotations with significantly different relative frequencies between phytopathogenic and non-phytopathogenic species, and with IPR annotations shared with PHI-base genes are shown. Z-scores were calculated using percentages of total annotations for a strain. Two lines track the mean Z-scores for *A. flavus* and *A. fumigatus*. Similar graphs for GO and KEGG annotations are shown in Additional file 8. Annotation terms and definitions are listed in the same order as the x-axes in Additional file 9

Differentially-assigned annotations (DAAs) were defined as annotations with significantly different relative frequencies between phytopathogenic and non-phytopathogenic strains within a set of InterPro (IPR), Gene Ontology (GO) or Kyoto Encyclopedia of Genes and Genomes (KEGG) annotation terms. Most (80.8 to 91.5%) of the AUGUSTUS-predicted genes in each strain were assigned at least one functional annotation term (Additional file 5). Of the retrieved 211 genes from PHI-base, 209 were annotated with 325 IPR, 322 GO and 379 KEGG terms (Additional file 7). Between the phytopathogenic and non-phytopathogenic species, 316 IPR, 214 GO and 1603 KEGG terms were found to have significantly different relative frequencies of assignment. Among these, 58 IPR, 51 GO terms and 74 KEGG terms matched those applied to *Aspergillus* genes in the PHI-base database. Limiting the search to only functionally annotated PHI-base genes that non-lethally affect pathogenicity and/or virulence of *Aspergillus* when mutated yielded 32 IPR, 20 GO terms and 52 KEGG DAAs (Fig. 2, Additional files 8, 9). The matched IPR DAAs that were higher in phytopathogenic strains relative to number of predicted genes included annotations related to fatty acid synthesis (IPR026025), oxidoreduction (IPR036812, IPR023210), zinc permease (IPR004698), secondary metabolite synthesis (IPR001227, IPR001242, IPR010071, IPR014030, IPR016035, IPR020801, IPR020841, IPR032088, IPR042099) and pectin degradation (IPR011050). Matched IPR annotations assigned more frequently to the non-phytopathogenic species included phosphatases and kinases (IPR000719, IPR011009, IPR036457), and sugar hydrolases and transferases (IPR001830, IPR017853). Multiple amino acid and sugar metabolism IPR annotations that were not shared between the list of DAAs and PHI-base gene annotations were overrepresented in *A. fumigatus* compared to *A. flavus*. These 21 terms predicted a chitinase, a galactose mutarotase, glycoside hydrolases, glucoamylases and a peptidoglycan deacetylase (Additional file 10, highlighted in blue).

DAA GO and KEGG terms related to peptidoglycan metabolic processes (GO:0009254), glycosaminoglycan metabolic processes (GO:0030203), carbohydrate metabolic processes (GO:0005975), peptidoglycan turnover (GO:0009254), amino sugar and nucleotide sugar metabolism (KO00520), and galactose metabolism (KO00052) were commonly enriched in the non-phytopathogenic strains *A. fischeri*, *A. flavus* and *A*. *sydowii*, but not in *A. nidulans*. Except for *A*. *terreus*, terms related to phenol-containing compound metabolic processes (GO:0018958) and histidine metabolism (KO00340) were enriched in the phytopathogenic strains. Further, *A. flavus*, *A*. *oryzae*, *A. parasiticus*, *A*. *sojae* and *A*. *tamarii* had enrichment of a glycosaminoglycan degradation term (KO00531).

**Fig. 3:**
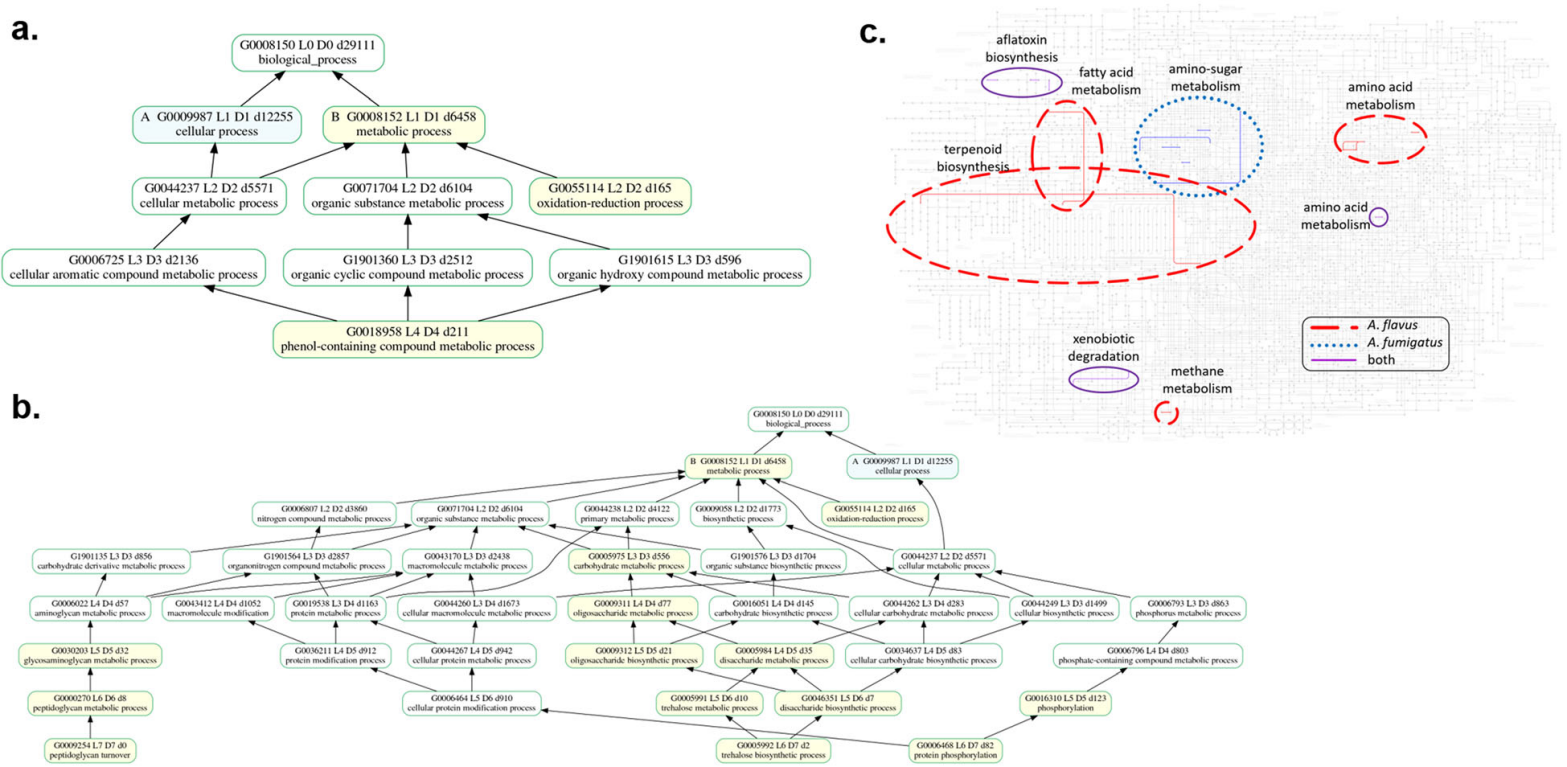
Enrichment of GO and KEGG annotation terms. Enrichment of GO biological process terms for **a**. *A. flavus* and **b**. *A. fumigatus*, and **c**. KEGG pathways for both species on the KEGG Mapper map for *A. flavus* metabolic pathways. Enrichment was based on sub-proteomic annotations compared to annotations of the full predicted proteomes. Enriched GO terms are in yellow-shaded boxes. Enriched KEGG pathways are highlighted in the indicated colors. The original diagram of *A. flavus* KEGG metabolic pathways is shown in Additional file 11

The major difference between the phytopathogens and non-phytopathogens was the number of translated amino acid sequences annotated to a broad category of amino and/or sugar (amino-sugar) metabolism and modification terms. This trend was also observed when comparing the enrichments between the full proteomes and sub-proteomes of *A. flavus* NRRL 3357 and *A. fumigatus* Af293. The strains had 226 and 131 proteins, respectively, back-matched from the DAAs. Sub-proteomic enrichment analyses identified many enriched GO and KEGG terms (Fig. 3; Additional file 11). *A. flavus* NRRL 3357 uniquely had enrichment of arylsulfatase activity (GO:0004065), transferase activity (GO:0016603, GO:0016755), 121 polyketide and secondary metabolite synthesis terms (Additional file 10, highlighted in orange) and fatty acid synthases (K00665). The sub-proteomic annotation of *A. fumigatus* Af293 was enriched in functions related to amino-sugar metabolism (GO:0000270, GO:0009254, GO:0030203, K00844, K12407), carbohydrate metabolism (GO:0005975, GO:0005984, GO:0005991, GO:0005992, GO:0009311, GO:0009312, GO:0046351), phosphorylation (GO:0004672, GO:0006468, GO:0016310, GO:0016772, GO:0016773, K02216, K07198, K08794, K08811), nucleotide binding (GO:0000166, GO:0005524, GO:0017076, GO:GO:0030554, GO:0032553, GO:0032555, GO:0032559, GO:0035639, GO:0097367, GO:1901265, K11665), oxidation-reduction (K22727, K22728), steroid biosynthesis (K00512, K21445, K22726) and chromatin structure (K14440).

Annotation terms related to amino-sugar metabolism IPR037950, GO:0000270, GO:0009254, GO:0030203, K00844, K12407, K13748, K19223 and K21471 were assigned to 12 *A. flavus* NRRL 3357 and 17 *A. fumigatus* Af293 AUGUSTUS-predicted protein-coding genes. These predicted *A. flavus* and *A. fumigatus* genes were then matched to 9 and 17 NCBI-curated proteins of *A. flavus* NRRL 3357 (XP_002372155.1, putative hexokinase; XP_002372244.1, putative hexokinase; XP_002373069.1, integral membrane protein; XP_002373361.1, polysaccharide deactylase family protein; XP_002373849.1, hexokinase family protein; XP_002375551.1, hexokinase family protein XprF; XP_002379109.1, putative oxidoreductase; XP_002382200.1, putative hexokinase Kxk; XP_002385204.1, UPF0075 domain protein) and *A. fumigatus* Af293 (XP_746328.1, UPF0075 family protein; XP_747575.1, polysaccharide deactylase family protein; XP_747679.1, putative hexokinase; XP_747854.1, putative glucokinase GlkA; XP_747946.1, putative LysM domain protein; XP_748231.1, hypothetical protein AFUA_5G0110; XP_749104.1, putative hexokinase; XP_749181.1, putative hexokinase; XP_749720.1, putative hexokinase Kxk; XP_751374.1, putative C6 transcription factor; XP_752897.1, polysaccharide deactylase family protein; XP_753281.1, putative NlpC/P60-like cell-wall peptidase; XP_753725.1, polysaccharide deactylase family protein; XP_755146.1, hexokinase family protein; XP_755905.1, putative hexokinase family protein XprF; XP_755969.1, putative glucokinase; XP_001481464.1, hypothetical protein AFUA_6G09315), respectively.

### A hexokinase gene is upregulated in response to co-inoculation with human cells

**Fig. 4:**
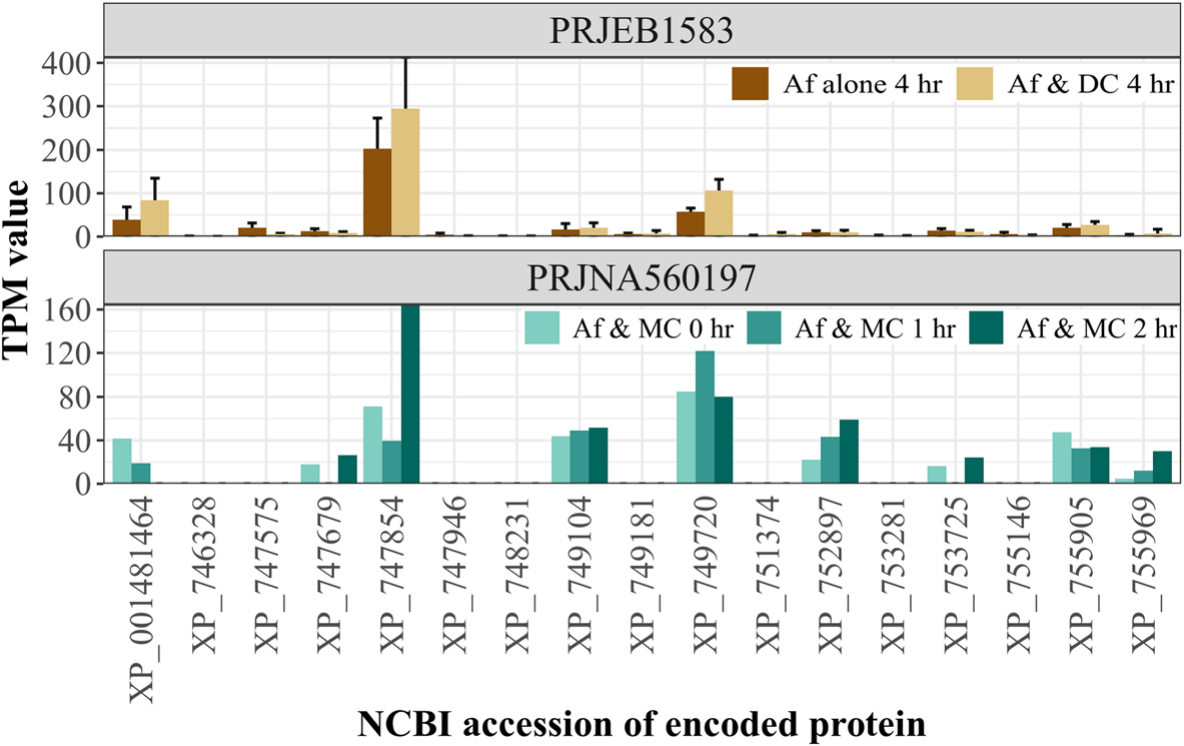
Gene expression during co-culture with human immune cells. Expression of *A. fumigatus* (Af) amino-sugar processing genes during co-culture with dendritic cells (DC) for 4 h or macrophages (MC) for 0, 1 or 2 h. RNA transcripts were quantified as transcript per million reads (TPM) values for the 17 *A. fumigatus* Af293 genes of interest. Error bars represent one standard deviation above the mean. Facets are labeled by the NCBI SRA Project accession. The gene encoding XP_749720.1, a hexokinase, was significantly up-regulated during co-culture with dendritic cells

The percentages of sequencing reads from NCBI SRA accession PRJNA560197 that mapped to the *A. fumigatus* genome were low, probably reflecting lower fungus to human ratios in the co-cultures compared to the experiments performed to yield the reads for the PRJEB1583 dataset (Additional file 12). For the former, sequencing reads mapped to 48.8 to 66.5% of *A. fumigatus* NCBI-annotated genes; the PRJEB1583 reads mapped to 93.5 to 98.1% of the genes. One gene, encoding the hexokinase XP_749720.1, was significantly (*p*-value = 0.0006) up-regulated when *A. fumigatus* was co-cultured with human dendritic cells for 4 h compared to incubation alone (Fig. 4). The gene also had increased expression when the fungus was cultured in a similar nutrient medium with macrophage-like cells for 1 h. However, at 2 h, the gene expression lowered back to the initial level. Non-significant upregulation was commonly observed for the gene encoding the glucokinase XP_747854 (*p*-value = 0.18). Genes encoding these proteins were not in the PHI-base.

## Discussion

The aim of this study was to identify candidate protein-coding genes within the *Aspergillus* genus that may contribute to pathogenicity. A comparative protein annotation approach was employed in which differential frequencies of annotation assignments were used to find predicted annotations that may be over or underrepresented (DAAs) within a set of genomes. The structural annotations did not yield a meaningful distinction between the phytopathogenic and non-phytopathogenic *Aspergillus* strains, though the larger number of secondary metabolite gene clusters in the phytopathogenic species tracked with the fact that fungal plant pathogens have larger secretomes than non-phytopathogens [26]. In contrast, all three sets of IPR, GO and KEGG functional annotations indicated that genes functioning in amino-sugar metabolism were assigned relatively more frequently to genes of the non-phytopathogenic strains *A. fischeri*, *A. fumigatus* and *A*. *sydowii*. Six of the *A. fumigatus* predicted genes retrieved from PHI-base were predicted to be involved in sugar metabolism, being annotated as glycosyl transferases, glycoside hydrolases, glucan synthases or mannosidases. Five of these genes cause loss of virulence or death when mutated: *FKS1* (PHI-base accession PHI2533), *AGS1* (PHI3902), *AGS2* (PHI3903), *AGS3* (PHI3904) and *GEL2* (PHI434) [27–29]. One gene, *tslA* (PHI7121; trehalose synthase), increases virulence after mutation [30]. The one carbohydrate metabolism-related *A. flavus* PHI-base gene (*PECA*; PHI88) was a pectinase [31].

The agreement among predicted overrepresented annotations led to the hypothesis that the identified genes play important roles in the pathogenicity of *A. fumigatus*, which might be indicated by differential expression during infection and disease progression in human hosts compared to growth in single-species cultures. Briefly, invading *A. fumigatus* is subject to phagocytosis by macrophages mediated by antigen-presenting dendritic cells. Analyses of publicly available RNA-Seq datasets indicated that one *A. fumigatus* Af293 hexokinase gene encoding the protein XP_749720.1 out of 17 observed candidates has time-dependent increased expression when the fungus is incubated with human immune cells. This gene is not known to have been previously studied as a pathogenicity factor. Its nucleotide sequence did not match (E-value ≤10) any human genes in the NCBI database, indicating that human RNA sequencing reads extracted from co-cultures should not substantially map to this hexokinase gene in the *A. fumigatus* genome. If the hexokinase is a pathogenicity factor and has a sufficiently different structure from human proteins, it may be useful as a target for inhibitory drugs to treat aspergillosis.

While it is not immediately clear what roles sugar-metabolizing genes may play in aspergillosis caused by *A. fumigatus* beyond energy production and storage, it can be hypothesized that the genes are involved in fungal pathogen signaling on the cell wall. Pathogenic fungi interact with host immune systems via chemical signatures called pattern-associated molecular patterns (PAMPs) and host pattern recognition receptors. Fungal PAMPs include complex carbohydrates in the cell walls which bind Toll-like receptors and C-type lectin receptors found on animal mononuclear phagocytes [32–36]. This binding initiates signaling cascades that induce the release of cytokines, phagocytosis and cell death. Most fungal PAMPs in mammalian hosts are glucose-containing macromolecules, including mannoproteins, chitin and β-glucans [37].

The higher number of genes functioning in amino-sugar processing could also be related to *N*-acetylglucosamine synthesis and/or breakdown. This amino-sugar has structural and functional roles in fungal cell walls, and in cell signaling for expression of virulence genes of *Candida albicans* and pseudohyphal morphogenesis of *Candida* and *Yarrowia* species [38]. Hexokinases, specifically, are involved in morphogenesis and virulence along with their nominal roles in life-supporting sugar and *N*-acetylglucosamine metabolism in *A. fumigatus* and *Candida albicans* [39–42]. There are several reported disease-associated glycosylated antigens, detoxifying catalases and host-adhering sialic acids from *A. fumigatus* [10, 43–49]. *A. fumigatus* conidial surfaces have 3 to 20 times more sialic acids than the less virulent or non-pathogenic *Aspergillus* species *A. auricomus*, *A. ornatus* and *A*. *wentii* [50]. Perhaps, the *A. fumigatus* candidate pathogenicity factors identified here could be involved in the synthesis of those macromolecules. Fungal carbohydrates and bacterial peptidoglycans additionally induce plant innate immunity [51–53]. Altogether*,* the overrepresentation of amino-sugar processing genes in *A. fumigatus* compared to *A. flavus* may suggest that *A. fumigatus* PAMPs are modified in unique, species-specific ways to be misrecognized or induce improper responses by immunocompromised animal hosts and/or are easily recognized by plant hosts. Further experimental study is required to properly test the above hypotheses and to understand the differences among the strains used here.

While the authors are unaware of strain idiosyncrasies that may result in one strain being better classified as plant pathogenic or not, the strains had noticeable differences in genome characterizations. The genus itself is not amenable to neat categorization between phytopathogenic and non-phytopathogenic species. Differences in strain virulence and/or host preference may be implied by isolation source (Additional file 1). These differences may have been obscured here due to grouping by named species. The non-phytopathogenic species *A. fumigatus*, *A. nidulans* and *A*. *sydowii* can be endophytes [54–56]. Despite not being associated with plant diseases, *A. fumigatus* has genes encoding cellulases, hemicellulases and pectinases, but no gene sets uniquely shared with non-*Aspergillus* human fungal pathogens [57]. In this study, *A*. *terreus* was found to have a lower number of proteins annotated as pectinase-like proteins compared to other phytopathogenic species, which may reflect that *A*. *terreus* infects leaves where pectin levels are lower compared to pectin levels in fruits and seeds [58–60]. *A. nidulans* had fewer amino-sugar metabolism genes than the other non-phytopathogenic species. *A. nidulans*, compared to *A. fumigatus*, induces a weaker oxidative burst by human immune cells and is phagocytosed at a slower rate by rodent macrophages [61].

## Conclusions

A direct correlation between frequencies of amino-sugar processing genes and virulence in animal hosts by *Aspergillus* strains could support the hypothesis that the amino-sugar genes of interest are involved in pathogen-host recognition. An application of the comparative protein annotation method used here to additional transcriptomic or proteomic data would help further identify and test pathogenicity candidates by their expression patterns.

## Methods

### Genomes, gene predictions and annotations

Genomic sequences were retrieved from the NCBI Genome database [62]. Only species with at least three different strain genomes publicly available by April 14, 2020 were included, totaling 217 genomes from 12 species of *Aspergillus*: three from *A. fischeri*, 64 from *A. flavus*, 14 from *A. fumigatus*, three from *A. nidulans*, 17 from *A. niger*, 92 from *A*. *oryzae*, three from *A. parasiticus*, five from *A*. *sojae*, three from *A*. *sydowii*, three from *A*. *tamarii*, seven from *A*. *terreus* and three from *A*. *tubingensis* (Additional file 1). Strain *A*. *terreus* ATCC 20542 was excluded due to an abnormally low genome size (138.52 kbp). *Aspergillus* species were classified according to reported ability to cause disease and persistent rot in live plants in environmental settings. Therefore, *A. flavus*, *A. niger*, *A. parasiticus*, *A*. *tamarii*, *A*. *terreus* and *A*. *tubingensis* were labeled as phytopathogenic and were compared to the non-phytopathogenic species *A. fischeri*, *A. fumigatus*, *A. nidulans* and *A*. *sydowii*. *A*. *oryzae* and *A*. *sojae* comprise domesticated strains of *A. flavus* and *A. parasiticus*, respectively, and may not reflect natural loss of phytopathogenicity [63–65]. These two species were excluded from statistical tests comparing phytopathogenic to non-phytopathogenic species. Secondary metabolite gene clusters were predicted for all strains using antiSMASH at default settings [66].

Gene predictions were performed on both DNA strands using AUGUSTUS version 3.0 trained on *A. nidulans* for all *Aspergillus* strains [67]. The resulting genomic annotations for each strain were parsed to calculate gene counts, average gene length (from start codon to stop codon, inclusive), average exon frequency per gene, average exon length (inclusive of stop codon), average intron frequency per gene, average intron length, average gene GC content (from start codon to stop codon, inclusive), average exonic GC content (inclusive of stop codon), average intronic GC content and codon usage (exclusive of stop codons TAA and TAG). Translated sequences were functionally annotated using InterProScan version 5.39 and KofamScan version 1.2 [68, 69], and the frequencies of each assigned (E-value ≤1^− 50^) IPR, GO and KEGG annotation term were counted. Frequencies of annotations for each strain were normalized as percentages of total predicted gene count for the strain. The 211 *Aspergillus* genes present in PHI-base version 4.9 were uploaded after being experimentally studied as pathogenicity or virulence factors by other researchers [70]. The PHI-base set of genes comprised 18 from *A. flavus*, 189 from *A. fumigatus* and 4 from *A. nidulans*. These genes were retrieved and subjected to annotation by InterProScan and KofamScan.

### Identification of *A. flavus* and *A. fumigatus* proteins with enriched annotations

Two-way analysis of variance (ANOVA) was used to determine if species identity was a factor in multiple genomic structural aspects. DAAs were identified using PCA and independent *t*-tests. Statistical tests were performed with counts of assigned annotations normalized as percentages of total annotations for a strain. Figures were generated using R packages ape, DECIPHER, dplyr, factoextra, FactoMineR, ggdendro and ggplot2 [71–78]. For *t*-tests, relative differences of at least 0.01% with Benjamini-Hochberg corrected *p*-values < 1^− 4^ (false discovery rate = 0.1%) were considered significant. Excluding the non-hierarchical IPR terms, GOATOOLS and Fisher’s exact test (α-level = 1^− 3^ for GO annotations; 1^− 6^ for KEGG annotations) were used to identify enriched GO and KEGG terms compared to the full list of proteomic annotations [79]. DAAs between phytopathogenic versus non-phytopathogenic species were back-matched to the predicted proteomes to produce sub-proteomes. The derived proteins of interest had functional annotation terms only present in the list of DAAs. In other words, proteins with annotation terms not in the DAA list were excluded from the sub-proteomes. Enrichment analyses with GOATOOLS and Fisher’s exact test also were performed in a second alternative method, comparing the annotated sub-proteomes to the annotated full proteomes. GOATOOLS and KEGG Mapper were used to generate figures with the enriched terms [78, 79].

### Transcriptomic data analysis of *A. fumigatus* cultured with human immune cells

RNA-Seq reads were retrieved from the NCBI SRA database project accessions PRJEB1583 and PRJNA560197 [80, 81]. The datasets comprised RNA-Seq reads from *A. fumigatus* incubated alone or with human dendritic cells (PRJEB1583; read accessions ERR236917, ERR236920, ERR236932, ERR236939, ERR236940, ERR236942, ERR236948, ERR236949, ERR236951, ERR236953, ERR236959, ERR236962, ERR236963, ERR236972), or with macrophage-like cells (PRJNA560197; read accessions SRR9965307, SRR9965308, SRR9965309). The sequencing reads were processed as previously described with slight modifications [23]. Briefly, read quality was checked, then aligned to the *A. fumigatus* Af293 genome (NCBI GenBank assembly GCA_000002655.1) guided by the respective GFF3 file [82]. Utilizing BLAST, AUGUSTUS-predicted *A. fumigatus* Af293 proteins with enriched DAAs of interest were matched to accessions in the NCBI Protein and Nucleotide databases with a maximum E-value of 1^− 100^ [83]. Presence of the corresponding genes was assessed in all experimental groups: *A. fumigatus* spores incubated alone or co-cultured with human dendritic cells (5:1 spores:human cells) in complete RPMI 1640 medium at 37 °C for 4 h (PRJEB1583, seven replicates), and *A. fumigatus* cultured with a human leukemia cell line differentiated into macrophage-like cells (2:1 spores:human cells) for 0, 1 or 2 h at 37 °C in a modified RPMI 1640 medium (PRJNA560197, one replicate). For the PRJEB1583 experiment, *t*-tests (α-level = 1^− 3^) were performed to compare gene expression quantified as transcripts per million reads.

## Supplementary Information

**Additional file 1: Table S1.** Strains included in the present study.

**Additional file 2: Figure S1.** Structural aspects of predicted *Aspergillus* genes. **a.** Number of genes; **b.** average exon count per gene; **c.** average intron count per gene; **d.** average gene length; **e.** average exon length per gene; **f.** average intron length per gene; **g.** average gene GC content; **h.** average exon GC content; **i.** average intron GC content.

**Additional file 3: Figure S2.** Amino acid usage among predicted *Aspergillus* genes.

**Additional file 4: Figure S3.** Codon usage among predicted *Aspergillus* genes.

**Additional file 5: Table S2.** Overview of functional annotation of *Aspergillus* strains.

**Additional file 6: Figure S4.** Structural and functional annotation cluster by *Aspergillus* species. PCA and scree plots for **a., b.** structural aspects; **c.** IPR terms; **d., e.** GO terms; **f., g.** KEGG terms.

**Additional file 7: Table S3.** Functional annotation of pathogenicity/virulence *Aspergillus* genes.

**Additional file 8: Figure S5.** Relative frequencies of **a.** GO and **b.** KEGG terms associated with virulence in *Aspergillus*. Annotation terms and definitions are listed in the same order in Additional file 9.

**Additional file 9: Table S4.** Definitions of annotations shown in Fig. 2 and Additional file 8. Amino-sugar annotations mentioned in the text are highlighted in blue.

**Additional file 10: Table S5.** Differentially-assigned annotations not assigned to PHI-base genes. Overrepresented IPR amino-sugar terms are highlighted in blue. Enriched GO and KEGG polyketide and secondary metabolite synthesis terms are highlighted in orange.

**Additional file 11: Figure S6.** Original diagram of *A. flavus* KEGG metabolic pathways. Updates to the image are available via KEGG Mapper [79].

**Additional file 12: Table S6.** RNA-Seq metadata and read mapping results.

## Data Availability

Sources of genomic data and summarized results reported in the article are included in this published article and its additional files.

## Abbreviations

ANOVA: Analysis of variance
DAA: Differentially-assigned annotation
GO: Gene Ontology
IPR: InterPro
KEGG: Kyoto Encyclopedia of Genes and Genomes
PCA: Principal component analysis
