# Supplementary figures and images for: Identifying candidate Aspergillus pathogenicity factors by annotation frequency
Source: BMC Microbiol. 2020 Nov 11;20:342. doi: 10.1186/s12866-020-02031-y (PMC7661267; doi:10.1186/s12866-020-02031-y)

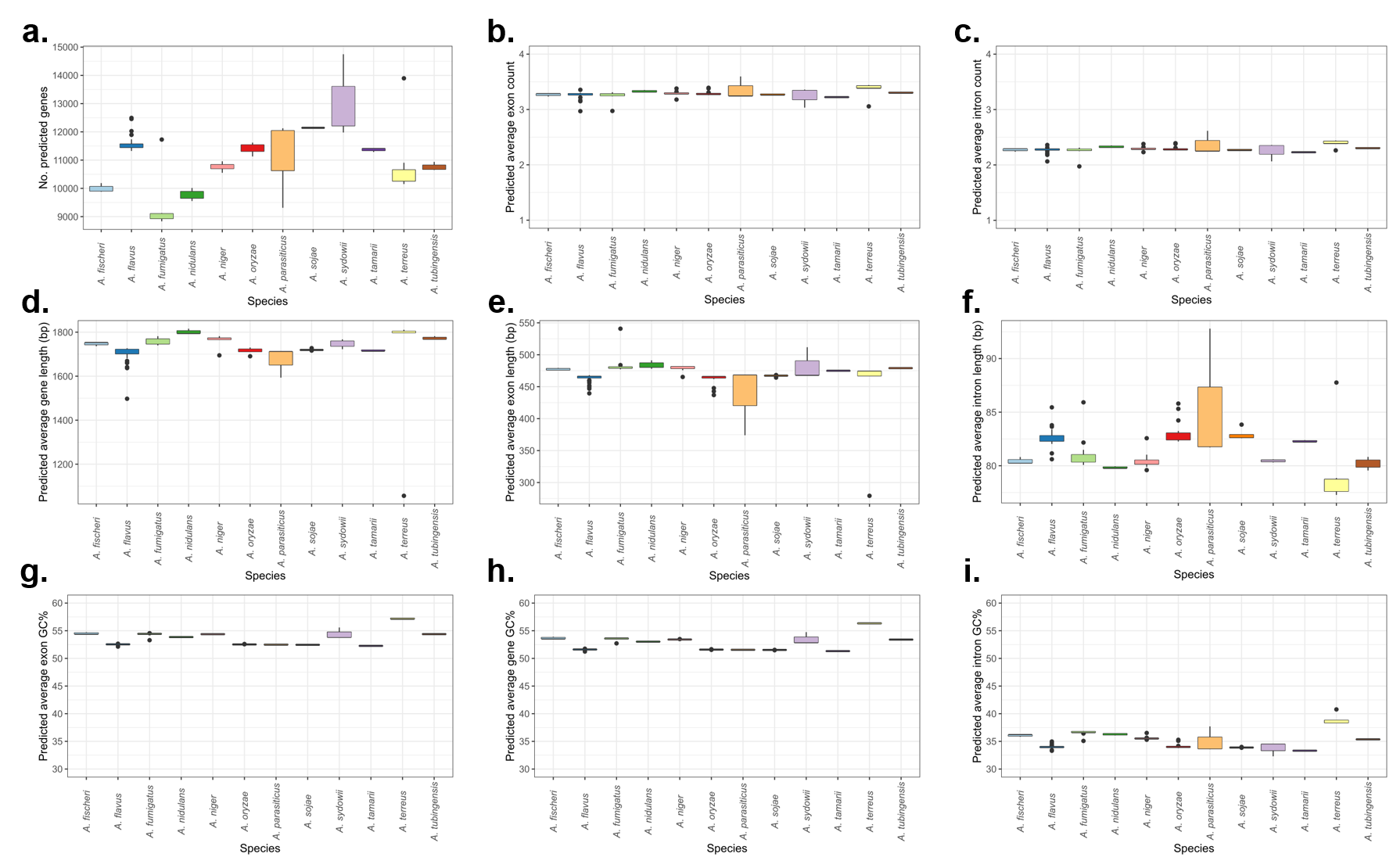

Supplement: Supplementary file 2 — Additional file 2: Figure S1. Structural aspects of predicted Aspergillus genes. a. Number of genes; b. average exon count per gene; c. average intron count per gene; d. average gene length; e. average exon length per gene; f. average intron length per gene; g. average gene GC content; h. average exon GC content; i. average intron GC content. [file 12866_2020_2031_MOESM2_ESM.png]

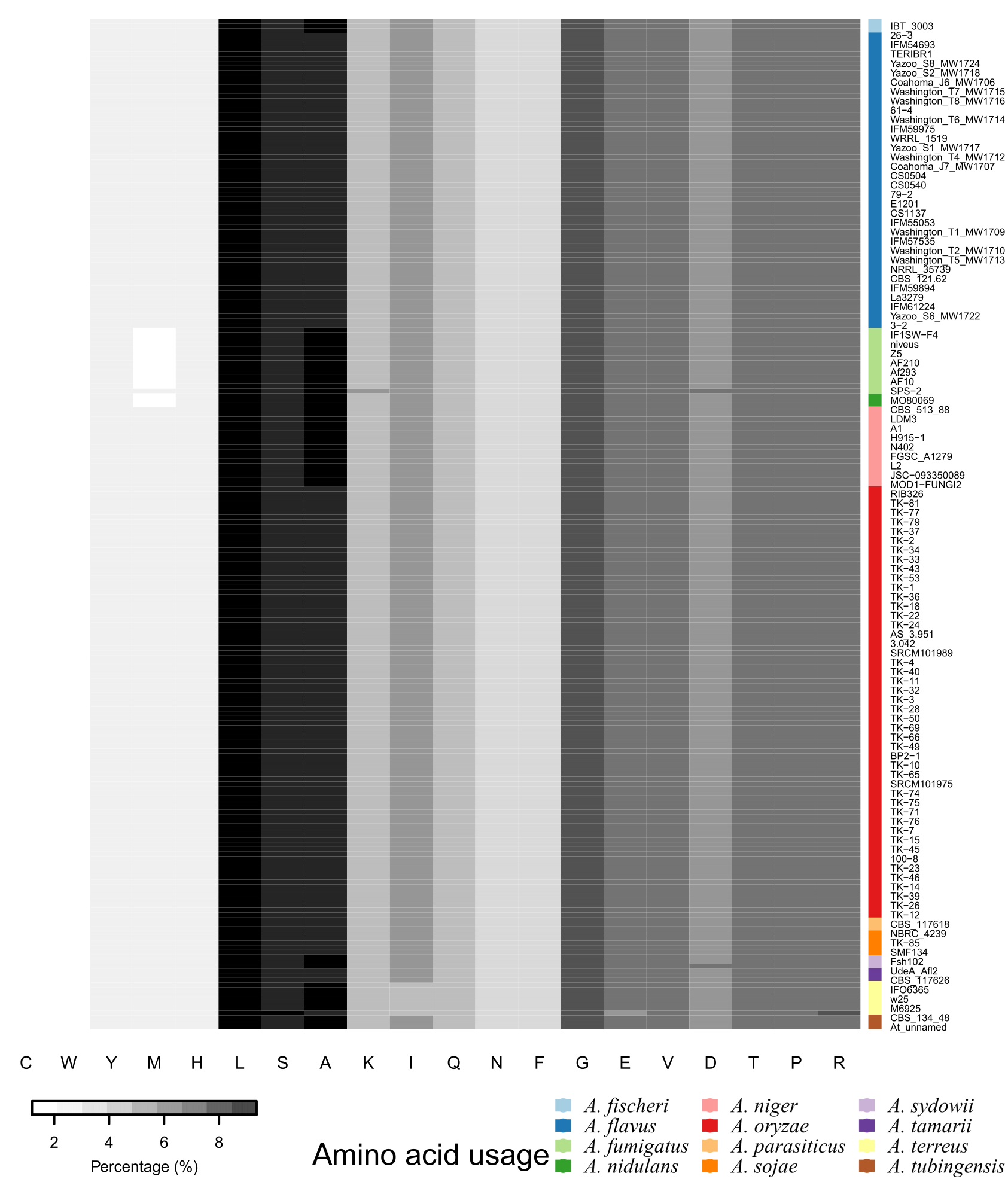

Supplement: Supplementary file 3 — Additional file 3: Figure S2. Amino acid usage among predicted Aspergillus genes. [file 12866_2020_2031_MOESM3_ESM.png]

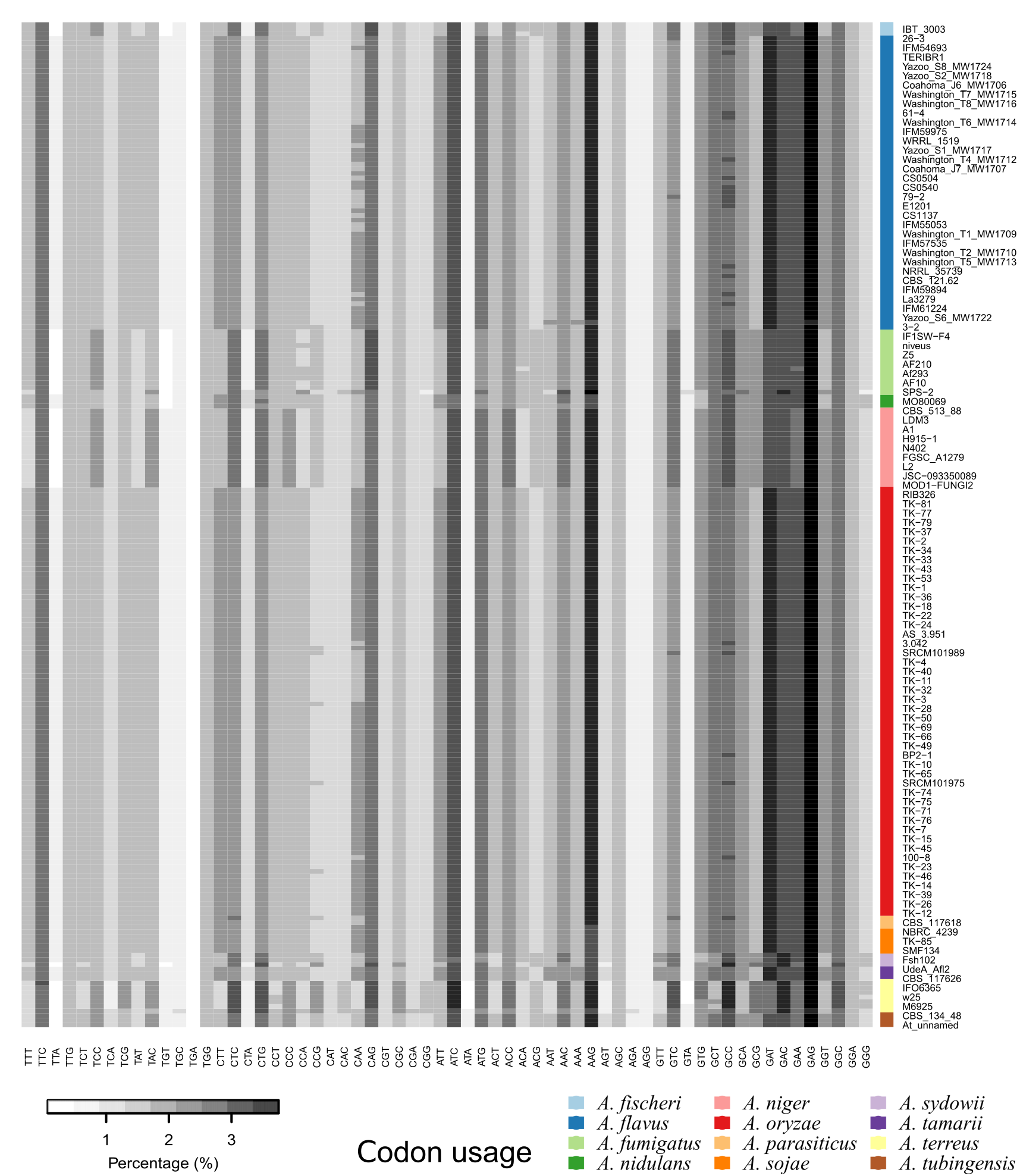

Supplement: Supplementary file 4 — Additional file 4: Figure S3. Codon usage among predicted Aspergillus genes. [file 12866_2020_2031_MOESM4_ESM.png]

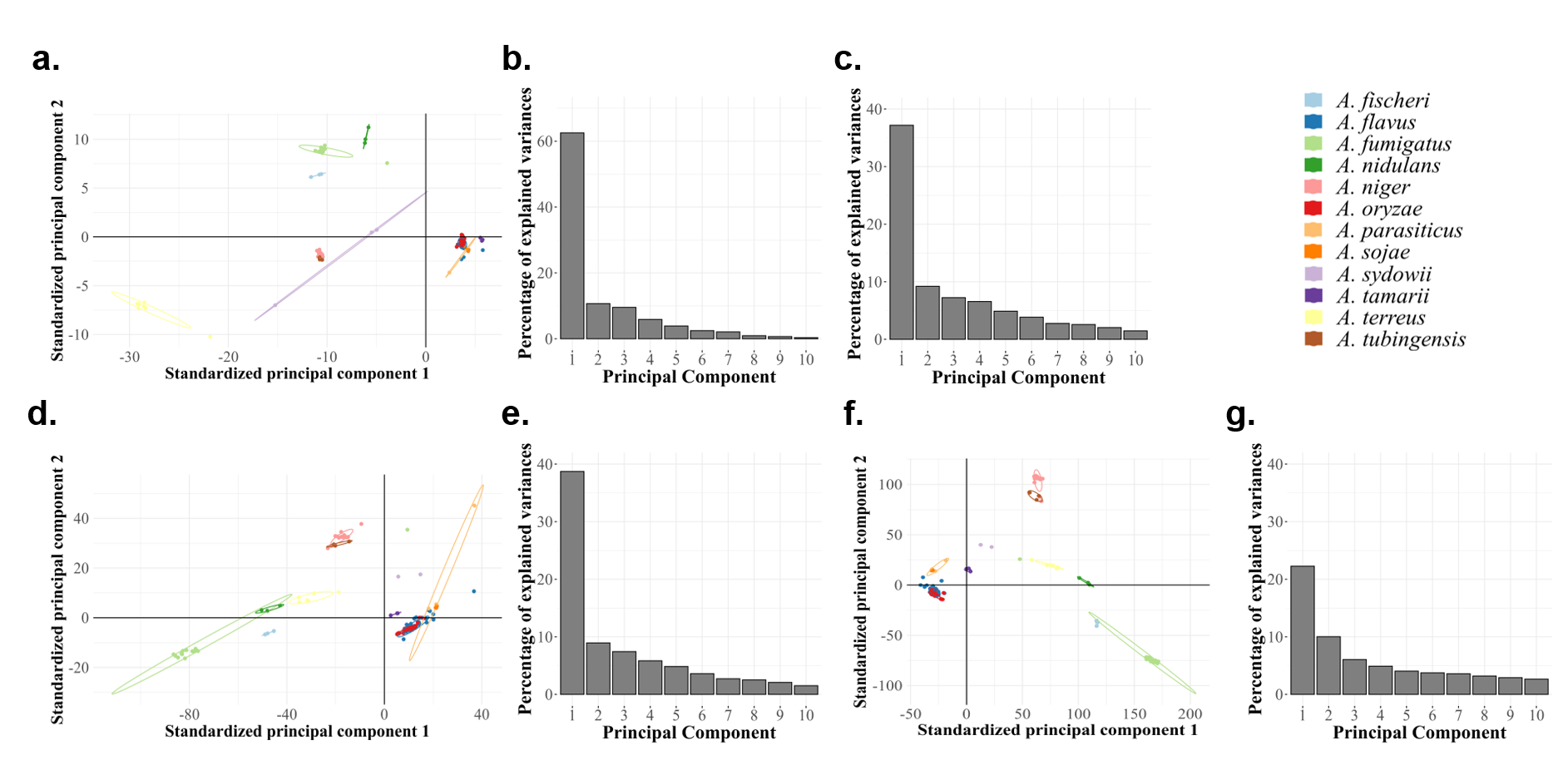

Supplement: Supplementary file 6 — Additional file 6: Figure S4. Structural and functional annotation cluster by Aspergillus species. PCA and scree plots for a., b. structural aspects; c. IPR terms; d., e. GO terms; f., g. KEGG terms. [file 12866_2020_2031_MOESM6_ESM.png]

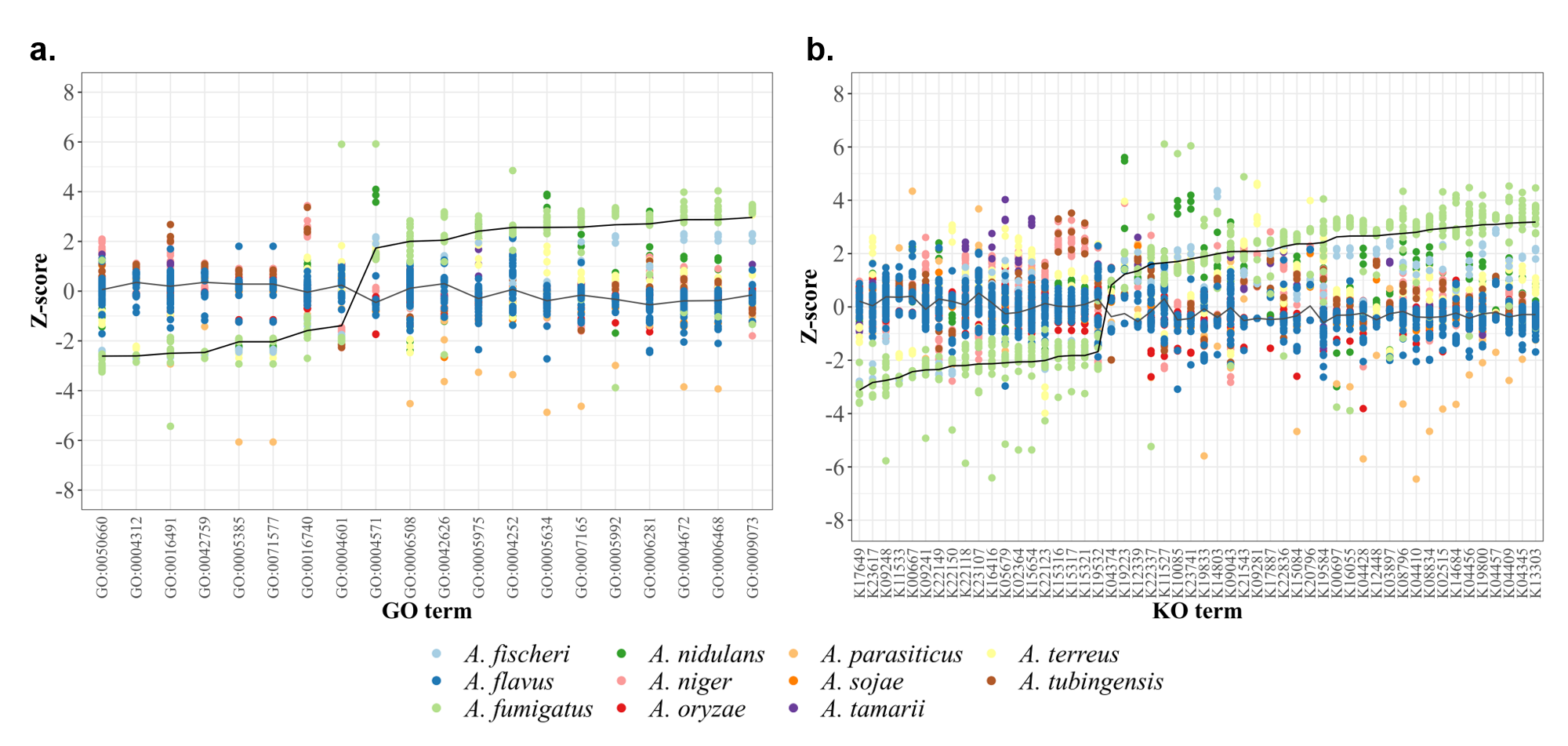

Supplement: Supplementary file 8 — Additional file 8: Figure S5. Relative frequencies of a. GO and b. KEGG terms associated with virulence in Aspergillus. Annotation terms and definitions are listed in the same order in Additional file 9. [file 12866_2020_2031_MOESM8_ESM.png]

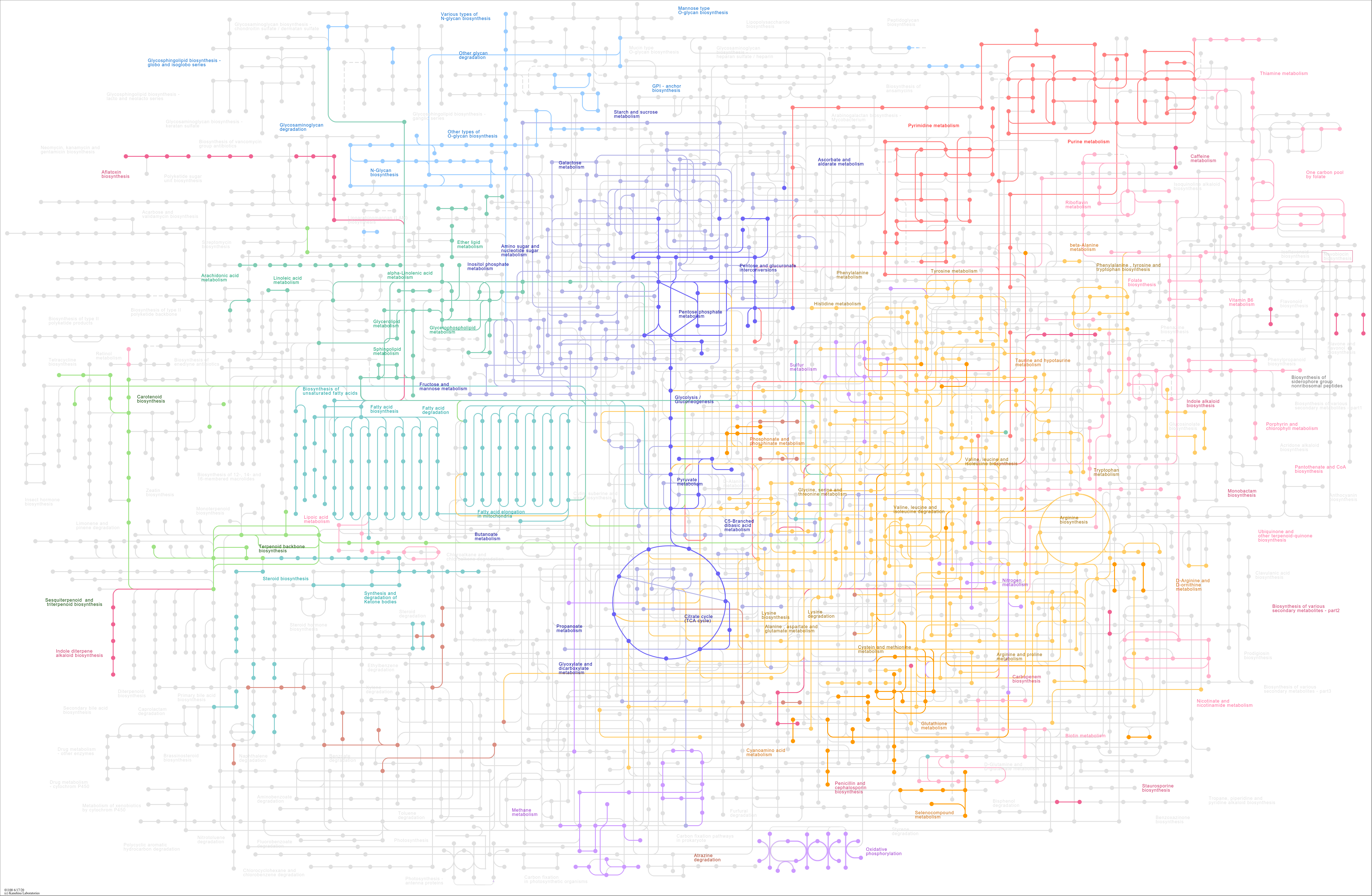

Supplement: Supplementary file 11 — Additional file 11: Figure S6. Original diagram of A. flavus KEGG metabolic pathways. Updates to the image are available via KEGG Mapper [79]. [file 12866_2020_2031_MOESM11_ESM.png]
